# Ubiquitin Ligases Involved in the Regulation of Wnt, TGF-β, and Notch Signaling Pathways and Their Roles in Mouse Development and Homeostasis

**DOI:** 10.3390/genes10100815

**Published:** 2019-10-16

**Authors:** Nikol Baloghova, Tomas Lidak, Lukas Cermak

**Affiliations:** Laboratory of Cancer Biology, Division BIOCEV, Institute of Molecular Genetics of the Czech Academy of Sciences, 252 42 Vestec, Czech Republic; nikol.baloghova@img.cas.cz (N.B.); tomas.lidak@img.cas.cz (T.L.)

**Keywords:** ubiquitin–proteasome system, cancer, mouse model, gene inactivation

## Abstract

The Wnt, TGF-β, and Notch signaling pathways are essential for the regulation of cellular polarity, differentiation, proliferation, and migration. Differential activation and mutual crosstalk of these pathways during animal development are crucial instructive forces in the initiation of the body axis and the development of organs and tissues. Due to the ability to initiate cell proliferation, these pathways are vulnerable to somatic mutations selectively producing cells, which ultimately slip through cellular and organismal checkpoints and develop into cancer. The architecture of the Wnt, TGF-β, and Notch signaling pathways is simple. The transmembrane receptor, activated by the extracellular stimulus, induces nuclear translocation of the transcription factor, which subsequently changes the expression of target genes. Nevertheless, these pathways are regulated by a myriad of factors involved in various feedback mechanisms or crosstalk. The most prominent group of regulators is the ubiquitin–proteasome system (UPS). To open the door to UPS-based therapeutic manipulations, a thorough understanding of these regulations at a molecular level and rigorous confirmation in vivo are required. In this quest, mouse models are exceptional and, thanks to the progress in genetic engineering, also an accessible tool. Here, we reviewed the current understanding of how the UPS regulates the Wnt, TGF-β, and Notch pathways and we summarized the knowledge gained from related mouse models.

## 1. Introduction

As revealed by the analysis of The Cancer Genome Atlas (TCGA), the Wnt, transforming growth factor-β (TGF-β), and Notch signaling pathways belong among the ten evaluated and curated canonical signaling pathways that are altered in most cancers [1]. It is the central role in governing and controlling cell proliferation which makes these pathways as well as their regulators vulnerable to cancer-associated somatic mutations [2,3,4]. The ubiquitin–proteasome system (UPS)-dependent regulation of Wnt, TGF-β, and Notch signaling is well known and established. Importantly, it represents a gateway for therapeutic modification and micromanagement of these signaling pathways, especially in the context of cancer [5,6]. To understand and exploit these possibilities it is necessary to evaluate the knowledge in vivo. Thus, the ambition of this review was to summarize the current understanding of how the UPS regulates Wnt, TGF-β, and Notch signaling. Additionally, we wanted to highlight the physiological roles of the ubiquitin ligases responsible for these regulations as they have been reported from currently available mouse models. Of note, the role of deubiquitinases (DUBs) was out of the scope of this text and is reviewed elsewhere [7].

## 2. The Ubiquitin–Proteasome System

**Figure 1:**
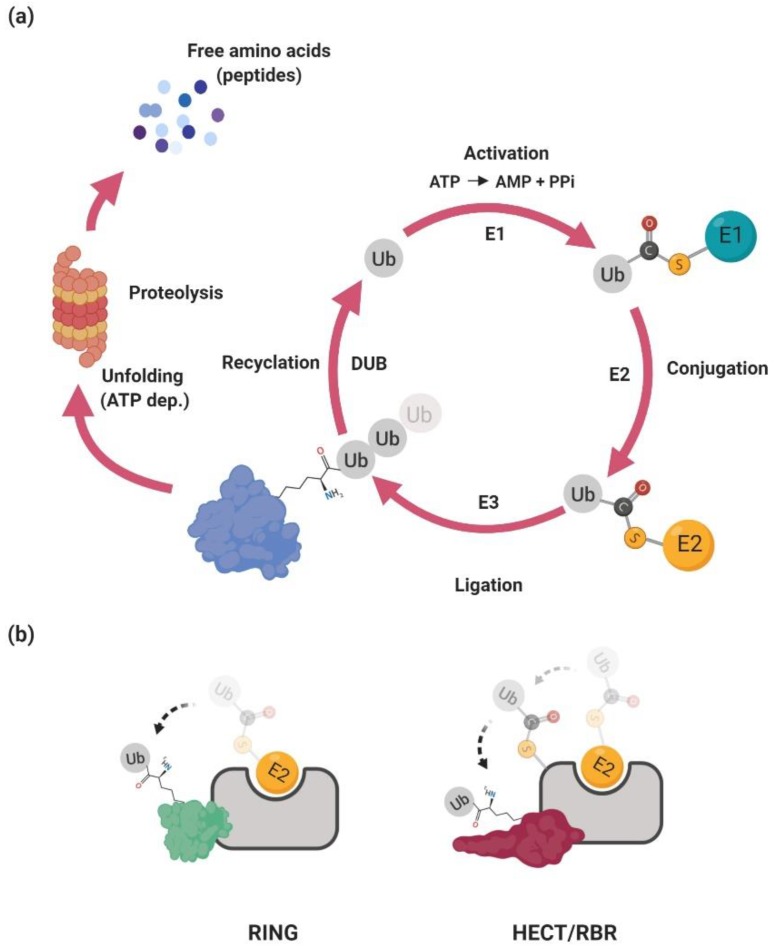
The ubiquitin–proteasome system. (**a**) The mature free ubiquitin monomer protein is either recycled from the ubiquitinated substrate or cleaved from the polyubiquitin precursor. Both of these reactions are catalyzed by deubiquitinases (DUBs). Ubiquitin is then activated (E1), conjugated (E2), and finally ligated to the cognate substrate via ubiquitin ligases (E3). The polyubiquitinated substrate is later transferred to the proteasome, unfolded, and proteolytically degraded to small peptides or free amino acids. For more details see the text. (**b**) RING E3s catalyze the direct transfer of ubiquitin from E2∼ubiquitin to the substrate. HECT (homologous to E6AP C-terminus), and RBR (RING-between-RING) E3s accept ubiquitin from E2 to form an E3∼ubiquitin thioester intermediate via transthiolation reaction. For more details see the text.

The ubiquitin–proteasome system regulates many cellular processes, including cell cycle, differentiation, DNA repair, and the immune response (for a review, see Reference [8]). Its main function is to achieve the precise temporal and spatial expression of a diverse repertoire of proteins. Essentially, the UPS delivers unneeded or damaged proteins to the proteasome where they are unfolded and ultimately chopped into small peptides. At a molecular level, the proteasome is a multisubunit protein complex with a central hollow part involved in the proteolysis and two proximal parts involved in the recognition of the substrate and its ATP-dependent unfolding. The selectivity of the UPS is accomplished by specific recognition of the target protein (i.e., substrate) which has to be covalently modified by a chain of small protein ubiquitin—polyubiquitinated (Figure 1a).

### 2.1. Ubiquitination

The ubiquitination is achieved via an enzymatic cascade in which ubiquitin is activated by covalent linkage to the E1 ubiquitin-activating enzyme. This activation is dependent on ATP-driven adenylation of ubiquitin followed by the covalent association of ubiquitin with the E1 enzyme via a thioester bond and subsequent transfer of the activated ubiquitin to the E2 ubiquitin-conjugating enzyme through trans-thioesterification [9]. In the final step, the E3 ubiquitin ligase mediates the transfer of ubiquitin to the lysine residue in the substrate [10]. The covalently linked ubiquitin then serves as an acceptor for another ubiquitin molecule, ultimately producing long polyubiquitin chains. The polyubiquitin chains can be linked via different lysine residues in the ubiquitin. At least for certain E3 ubiquitin ligases the type of polyubiquitin chain seems to be dependent on the different usage of the specific E2 enzyme [11]. Currently, there are more than thirty genes in human genome encoding proteins harboring E2 activity [12]. Some of them specifically modify proteins with linear ubiquitin chains (e.g., lysine 63–K63) that are not recognized by the proteasome but are involved in cellular signaling [13]. Others (e.g., K48, K11) are involved in proteasome-dependent degradation or their role is currently not clear (e.g., K27) [14]. Notably, a key regulatory step of the ubiquitination reaction is dictated by the E3 ubiquitin ligase, which determines substrate selection and a choice of polyubiquitin chain.

### 2.2. Ubiquitin Ligases

**Figure 2:**
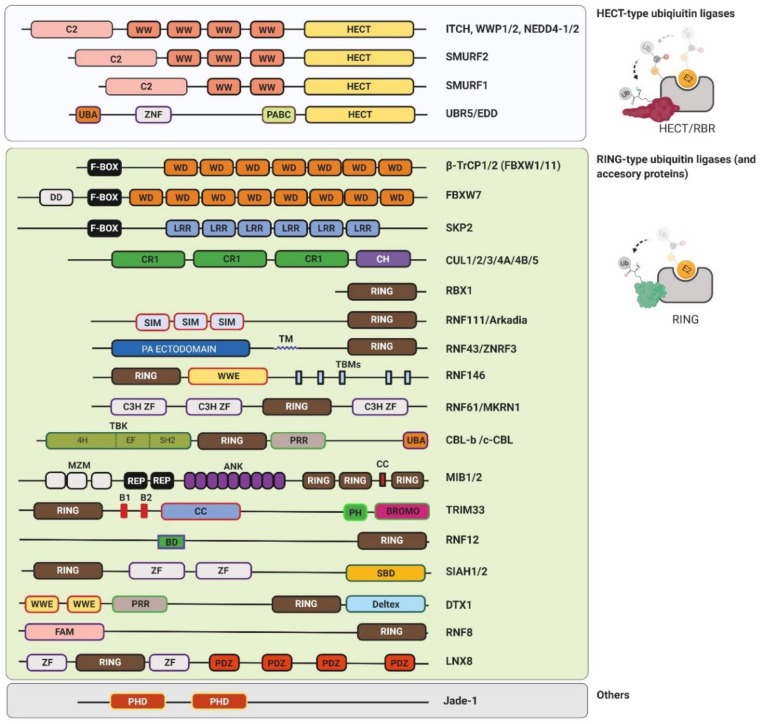
The modular structure of ubiquitin ligases involved in the Wnt, TGF-β, and Notch pathways.

The human genome encodes between 600–1000 E3 ubiquitin ligases. They are responsible for substrate selection and coordination towards the E2 enzyme (for a review, see References [15,16]). Currently, there are four main classes of ubiquitin ligases classified on the basis of the functional and structural features: RING (really interesting new gene), U-box, HECT (homologous to E6AP C-terminus), and RBR (RING-between-RING) [9]. The RING E3s catalyze the direct transfer of ubiquitin from E2∼ubiquitin to the substrate. The HECT and RBR E3s harbor a catalytic cysteine residue in their structure that accepts ubiquitin from E2 to form an E3∼ubiquitin thioester intermediate via transthiolation reaction (Figure 1b and Figure 2). This step is followed by the transfer of ubiquitin to the substrate lysine via aminolysis reaction.

The E3 ligases recognize the cognate substrates using different mechanisms. The specificity of ubiquitination is achieved via protein–protein interaction between a ubiquitin ligase and a substrate. This interaction can be direct or indirectly mediated by a protein or a small molecule adapter. The direct interaction is usually regulated by post-translational modification of the substrate or the ubiquitin ligase. In certain cellular processes, ubiquitin ligases have to be locally enriched to effectively bind and mediate substrate ubiquitination. A typical example is the RING finger 8 (RNF8) ubiquitin ligase, which is sequestrated at DNA damage sites where it targets a diverse spectrum of proteins (including histones) [17,18].

#### 2.2.1. RING-Type Ubiquitin Ligases

The RING ubiquitin ligases constitute two main classes based on the number of subunits (for a review, see References [6,19]). Monosubunit RING ligases form homo- and heterodimers or act as monomers. A typical example of these ubiquitin ligases is the mouse double minute 2 homolog (MDM2) protein. In the absence of DNA damage, MDM2 binds the p53 tumor suppressor and mediates its ubiquitination, with consequent proteasomal degradation [20]. The DNA damage-activated Ataxia-telangiectasia mutated (ATM) kinase inhibits MDM2–p53 interaction leading to p53 stabilization and activation of p53-dependent DNA damage response [21,22].

Multisubunit E3 ubiquitin ligase complexes, such as Cullin-RING ligases (CRLs), mediate ubiquitination of numerous substrates via variable substrate recognition modules [19]. They represent a dominant group of ubiquitin ligases. In mammals, there are eight different cullins which associate with large numbers of adaptor proteins, forming more than 200 CRLs. Many of these ubiquitin ligases are deregulated in a wide range of disorders including cancer and autoimmune syndromes [6,23,24].

#### 2.2.2. U-box-Type Ubiquitin Ligases

A specific group of RING-type ubiquitin ligases is U-box, containing ubiquitin ligases (for a review, see Reference [25]). A U-box is a 70 amino acid long domain with a similar fold as the RING domain. In contrast to RING, the U-box domain lacks conserved cysteine residues and it is not coordinated with zinc atoms. Nevertheless, the molecular mechanism underlying ubiquitination is similar for both U-box and RING-type ubiquitin ligases.

A typical example of U-box-type ubiquitin ligase is the C terminus of the Hsc70-interacting protein (CHIP) [26]. Upon heat stress, CHIP recognizes its substrates in the context of activated heat shock proteins, controlling the stability and fate of misfolded proteins.

#### 2.2.3. HECT-Type Ubiquitin Ligases

The human genome encodes 28 HECT ubiquitin ligases (for a review, see Reference [27]). They are characterized by a modular structure which comprises the N-terminal substrate-binding domain and the C-terminal HECT domain [27]. The HECT domain contains two lobes connected by a flexible hinge loop. The N-terminal lobe binds E2∼ubiquitin and the C-terminal lobe harbors the catalytic cysteine involved in the transfer of ubiquitin to substrates lysines. There are three different HECT-type ligase families: the NEDD (neural precursor cell expressed, developmentally downregulated) family, characterized by the tryptophan-rich WW domain involved in recognition of the PY motif; the HERC (HECT and RCC domain) family, which contains the regulator of chromosome condensation (RCC) 1-like domains; and the HECT family, with a spectrum of different protein–protein interaction domains.

A typical example of the HECT-type ubiquitin ligase is Smad ubiquitination regulatory factor 2 (SMURF2) protein [28]. The SMURF2 protein is a HECT-type E3 ubiquitin ligase from the NEDD4 subfamily. The WW domain, located in the N-terminal part of SMURF2 ubiquitin ligase, recognizes PPxY (PY) motifs in SMURF–targeted substrates [29]. One of the SMURF2 substrates is a TGF-β receptor I (TGF-βRI) [30]. The SMURF2 protein binds this receptor indirectly via Small mothers against decapentaplegic 7 (Smad7), an inhibitor of TGF-β signaling. The SMURF2 interacts with Smad7 in the nucleus. The complex of SMURF2 and Smad7 is then translocated to the cytosol where it recognizes and ubiquitinates TGF-βRI. This represents the strong negative feedback necessary to control TGF-β signaling and its dynamics.

#### 2.2.4. RBR-Type Ubiquitin Ligases

There are 14 RBR E3s identified in the human genome (for a review, see Reference [31]). They all contain a RING1–IBR–RING2 motif. The RING1 domain interacts with E2∼ubiquitin and mediates the transfer of ubiquitin to the catalytic cysteine of the RING2 domain. It was shown that RING2 forms a thioester intermediate with the C terminus of ubiquitin in a HECT E3-like manner and, consequently, transfers ubiquitin on the lysines of the selected substrates. An example of an RBR ubiquitin ligase is Parkin [32]. Parkin is involved in the recognition of proteins on the outer mitochondrial membrane [33]. Upon stress exposure, Parkin mediates mitochondria ubiquitination and its clearance via mitophagy [34].

## 3. Wnt Signaling Pathway and Its Regulation by Ubiquitin Ligases

Wnt ligands are extracellular soluble proteins. They are secreted by a diverse spectrum of cells and they are instrumental in the regulation of cell identity, migration, and proliferation [35,36]. Genes encoding the components of the Wnt signaling pathway are often misregulated or mutated in human cancers, especially in tissues with fast cellular renewal (e.g., breast, intestine, skin, prostate or lung). This is due to the central role of the Wnt signaling pathway in stem cell recovery and progenitor cell pool formation.

**Figure 3:**
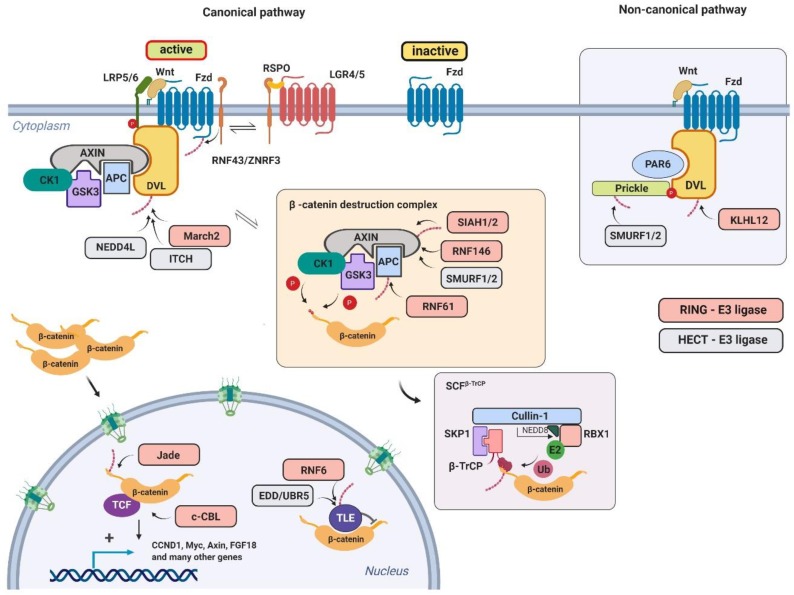
The Wnt signaling pathway and its regulation by ubiquitination The canonical Wnt signaling pathway is triggered by Wnt ligand binding to the complex of its receptor, Frizzled, and co-receptors LRP5/6. The activated receptor associates with the Dishevelled (DVL) protein and inhibits the β-catenin destruction complex. The stabilized β-catenin translocates and accumulates in the nucleus, where it activates the Wnt-dependent transcriptional program. For details see the text.

The Wnt signaling pathway is initiated upon Wnt binding to its cognate receptor. This is followed by the sequence of activation steps which lead to translocation of the β-catenin transcription co-activator to the nucleus (Figure 3). Nuclear β-catenin associates with DNA-binding transcriptional factors from the T-cell factor/lymphoid enhancer-binding factor (TCF/LEF) family and activates the Wnt-dependent transcriptional program.

In more detail, the intracellular signaling is triggered by Wnt binding to the complex of its receptor Frizzled (Fzd) and co-receptor from the lipoprotein receptor-related protein family (LRP5/6) [37]. When inactive, the Fzd protein level and its membrane localization are regulated negatively by two closely related ubiquitin ligases, RNF43 and Zinc–RING finger 3 (ZNRF3) [38,39]. These transmembrane ligases from the RING family interact with Fzd in the extracellular part and mediate ubiquitination of its cytosolic loops via the intracellular RING domain. This interaction is dependent on the intracellular protein Dishevelled, which (in the absence of Wnt stimulation) promotes Fzd degradation [40]. Upon the Wnt ligand engagement to Fzd, RNF43/ZNRF3 is inhibited by sequestration to the complex of leucine-rich repeat-containing G-protein coupled receptor (LGR4/5) transmembrane proteins. This interaction is mediated by a Wnt agonist from the R-spondin family [38].

The essential part of signal transduction initiated from the Fzd receptor relies on β-catenin destruction complex inhibition. This multisubunit complex consists of several regulatory and accessory proteins. Its major role is to mediate β-catenin phosphorylation by coordination of priming casein kinase 1 (CK1) and functionally redundant processing kinases glycogen synthase kinase 3α and 3β (GSK3α and GSK3β, respectivelly) [41,42,43]. Adapter proteins adenomatous polyposis coli (APC) and AXIN are responsible for this dynamic and precise phosphorylation machinery [44,45]. In detail, AXIN allows β-catenin Ser45 to be phosphorylated by CK1. This phosphorylation creates a docking site for GSK-3α/β, which subsequently phosphorylates β-catenin at Thr41, Ser37, and Ser33. The phosphorylated N-terminal part of β-catenin serves as a binding site (degron) for a β-transducin repeat-containing protein (β-TrCP) [46,47]. The β-TrCP is a canonical Cullin 1 (CUL1)-dependent F-box-containing substrate adapter. It associates via the S-phase kinase-associated protein 1 (SKP1) adapter and CUL1 scaffold proteins with the RING protein Ring-box 1 (RBX1). The RBX1 mediates β-catenin ubiquitination and degradation.

The Fzd activation by Wnt ligands results in Dishevelled-dependent oligomerization of LRP6 co-receptor and its CK1γ/GSK3-mediated phosphorylation [48,49]. In the AXIN-dependent manner, the cytosolic destruction complex is sequestrated to the membrane. This process inhibits the interaction of the destruction complex with β-catenin. Importantly, it was suggested that upon the activation of the Wnt signaling, the destruction complex is not inhibited per se. Rather, it leads to the inhibition of β-TrCP-dependent ubiquitination. Phosphorylated non-ubiquitinated β-catenin stays in the complex and blocks the association of newly translated molecules [50]. Stabilized (non-phosphorylated) β-catenin then translocates and accumulates in the nucleus, where it interacts with DNA-binding transcriptional factors from the TCF/LEF family [45]. The sustained Wnt signaling leads to the strong association of the β-catenin/TCF complex with target gene promoters, engagement of transcriptional co-activators, and, ultimately, to the activation of the Wnt-dependent transcriptional program.

The β-catenin destruction complex itself is a target for several ubiquitin ligases. The main substrate of the UPS-dependent regulation is the AXIN protein. The seven in absentia homolog 1 (SIAH1) ubiquitin ligase mediates ubiquitination of the AXIN protein [51]. The SIAH1 recognizes the AXIN VxP (Val-x-Pro) motif involved in AXIN–GSK3 interaction. The GSK3 counteracts SIAH-dependent AXIN ubiquitination, and, correspondingly, SIAH inactivation leads to the Wnt signaling attenuation. This regulatory process is important for sustained Wnt/β-catenin signaling. In a similar manner, poly ADP-ribosylated (PARylated) AXIN is a target of the ubiquitin ligase RNF146. PARylation of AXIN is dependent on the Tankyrase enzyme [52]. It was shown that RNF146 interacts directly with poly-ADP-ribose through its WWE domain and promotes degradation of many PARylated proteins [53,54]. The AXIN RNF146-dependent degradation seems to be dependent on a physiological context [55]. Both *Xenopus* and *Drosophila* models have shown the role of Tankyrase in AXIN degradation, but based on the findings from *Drosophila* studies, there seems to be redundancy on the RNF146 side [56,57]. HECT-type ubiquitin ligase, SMURF1, was shown to ubiquitinate the AXIN protein in a cell-cycle-dependent manner. Its interaction with AXIN is inhibited during G2/M which correlates with increased Wnt signaling [58]. The SMURF1-mediated AXIN ubiquitination does not lead to its degradation. Instead, Lys29-linked polyubiquitination of AXIN disrupts its interaction with the Wnt coreceptors LRP5/6, consequentially inhibiting Wnt signaling activation [59]. Close homolog of SMURF1, SMURF2, interacts with AXIN in a canonical WW-dependent manner. Ectopic expression of SMURF2 leads to AXIN protein level downregulation, and SMURF2 mediates AXIN ubiquitination in vitro [60]. The other subunit of the β-catenin destruction complex, APC, is also a target for the UPS. The RNF61 ubiquitin ligase, otherwise known as Makorin 1 (Mkrn1), binds to the armadillo repeats region of APC and targets it for proteasomal degradation. Inactivation of RNF61 leads to Wnt signaling inhibition, and this inhibition is rescued by concurrent APC knockdown [61].

The Dishevelled (Dishevelled 1–3) protein level is regulated by three other HECT-like ubiquitin ligases NEDD4L, NEDD4, and ITCH [62,63,64]. They were all shown to promote Dishevelled ubiquitination. Ubiquitin ligase NEDD4 positively regulates the maturation of cell–cell junctions in cooperation with the small GTPase Ras-related C3 botulinum toxin substrate 1 (Rac1). Activated Rac1 promotes Nedd4-mediated ubiquitination and degradation of Dishevelled 1 [64]. A close homolog of NEDD4, NEDD4L, attenuates Wnt/β-catenin signaling by regulation of Dishevelled 2 stability. The Wnt5a-induced c-Jun N-terminal kinase (JNK)-dependent phosphorylation of NEDD4L is critical for its activity towards Dishevelled 2 [62]. The inhibition of Wnt signaling via the ubiquitin ligase NEDD4L was observed in both *Xenopus* and human models [62,65]. The mammalian ortholog of *Drosophila* Suppressor of Deltex (Su(Dx)), ITCH, inhibits Wnt signaling upstream of β-catenin, by targeting activated Dishevelled 2 to proteasomal degradation [63]. The role of Dishevelled protein is not limited to the β-catenin destruction complex inhibition and its sequestration to the activated receptor. It is also involved in activation of the non-canonical pathway controlling planar polarity and proper tissue architecture. This pathway is β-catenin-independent and it is actively inhibited by ubiquitin ligase RNF43 and its interaction with Dishevelled protein. Transmembrane RING-type ubiquitin ligase RNF43 inhibits the non-canonical pathway in a ubiquitination-independent manner, and cancer-associated mutations of RNF43 do not have any effect on this activity [66]. Another E3 ubiquitin ligase promoting Dishevelled ubiquitination and degradation in the non-canonical pathway is the Cullin3-dependent substrate-binding adapter Kelch-like protein 12 (KLHL12). The KLHL12 binds Dishevelled in a Wnt-dependent manner, and KLHL12-dependent degradation of Dishevelled antagonizes the convergent extension movements of cells during gastrulation in zebrafish [67]. The study in the *Xenopus* model shows that another RING-type ubiquitin ligase membrane-associated ring-CH-type finger (MARCH2) is targeting Dishevelled during head development. The MARCH2 interaction with Dishevelled is dependent on the Dishevelled interaction partner Dapper1, and ubiquitinated Dishevelled is degraded in the lysosomal compartment [68].

Nuclear bound β-catenin is a target of several ubiquitin ligases. They mostly serve as crosstalk hubs from different pathways and signaling checkpoints involved in the control of the proper shutdown of the activated pathway. In hypoxic conditions, the Von Hippel–Lindau (VHL) tumor suppressor inhibits the Wnt pathway via promoting degradation of activated β-catenin. It is dependent on VHL-induced stabilization of the ubiquitin ligase Jade1 [69]. This is relevant for clear cell renal cell carcinomas (CCRCCs) with mutated VHL. The active VHL stabilizes Jade1 by interaction with its α- and β-domain. The stabilized Jade1 interacts with the β-catenin N-terminus and mediates its ubiquitination and degradation [70,71]. Of note, Jade1 does not belong to either HECT- or RING-type ligases. It contains two pleckstrin homology domain (PHD) fingers, and its intrinsic ubiquitin ligase activity has not yet been supported by independent observations. Casitas B-lineage lymphoma (c-CBL) is another ligase that binds preferentially to active β-catenin [72,73]. Wnt-dependent nuclear c-CBL seems to selectively inhibit pro-angiogenic Wnt effects [72].

One of the paradigms in Wnt signaling is that in the absence of Wnt stimulation, TCF/LEF factors are acting as transcription repressors and that this repression is abrogated by β-catenin binding. Mechanistically, TCF factors associate with repressors from the TLE (transducin-like enhancer) family in the absence of Wnt signaling, and this interaction is blocked by competitive binding between TLE repressors and activated β-catenin. The TLE factors are subsequently targeted by the E3 isolated by differential display/ubiquitin protein ligase E3 component n-recognin 5 (EDD/UBR5) ubiquitin ligase from the HECT family for ubiquitination and degradation [74]. Besides TLE factors, EDD ubiquitinates phosphorylated β-catenin, as well. Instead of degradation, it was observed that in the context of β-catenin, EDD promotes the growth of Lys29- and Lys11-linked ubiquitin chains, supposedly to potentiate β-catenin stability and signaling [75]. This pathway is probably redundant or cell context-dependent, as it was shown that in colorectal cancer it is rather the ubiquitin ligase RNF6 which promotes Wnt signaling via controlling the stability of the TLE3 factor [76].

The UPS-based quality control is another regulatory mechanism in the Wnt signaling pathway. This is important for endoplasmatic reticulum (ER)-associated protein production and restricted to secreted and transmembrane proteins. It was shown that in the absence of Wnt ligands, the cargo protein EVI is degraded via ER-associated degradation (ERAD) [77]. Additionally, the Wnt co-receptor LRP6 is targeted to the ERAD pathway as well [78,79]. Upon ubiquitination of its intracellular part, LRP6 presumably interacts with a ubiquitin-binding protein which acts as a chaperone for its correct folding. The successfully folded LRP6 is palmitoylated and transported to the cell surface [80]. If folding is impaired, another round of polyubiquitination targets LRP6 to the ERAD pathway.

The Wnt signaling pathway is also sensitive to common stress-inducers such as heat stress. Activation of heat shock proteins leads to activation of the U-box ubiquitin ligase CHIP, which is involved in ubiquitin-dependent clearance of misfolded proteins [81]. One of the substrates of CHIP ubiquitin ligase is β-catenin [82]. The recognition of β-catenin via CHIP rather represents a general mechanism involved in unfolded protein and heat shock response than specific regulation of the Wnt signaling pathway.

### 3.1. Mouse Models of Ubiquitin Ligases Involved in the Wnt Signaling Pathway

As mentioned above, Wnt signaling plays a crucial role in an array of developmental and homeostatic processes. Mouse models defective in the Wnt signaling pathway reflect this fact and display various defects. The phenotypes span from higher cancer incidence, stem cell depletion to defects in tissue polarity and anteroposterior patterning [45,83,84,85]. The role of several ubiquitin ligases that have been shown to regulate the Wnt signaling pathway in vitro was confirmed in the cognate mouse models. This was true for ZNRF3/RNF43, RNF146, RNF61, SMURF1/2, and partially for β-TrCP1/2 and Nedd4 ubiquitin ligases [39,55,86,87,88,89]. However, the mouse models of other ubiquitin ligases did not confirm the function in Wnt signaling and show either different or more general physiological functions. These observations probably arise from the fact that these ubiquitin ligases have other physiological substrates and control different processes. The other possible explanation is that the involvement of these ligases in physiological Wnt signaling is subtle. Therefore, these mouse models require more detailed analysis or challenges such as aging or stress response to show and confirm in vitro observations.

#### 3.1.1. β-Transducin Repeat-Containing Protein (β-TrCP)

Both β-TrCP1 and β-TrCP2 (also known as F-box and WD repeat domain, containing 1/11) are highly evolutionarily conserved F-box proteins [23]. They serve as substrate adapters for the CRL ubiquitin ligase. They both recognize a phosphodegron through seven WD-repeats assembled to a typical propeller structure. They are currently assigned to many different substrates including NFκB Inhibitor α (IκBα), β-catenin, and a canonical regulator of circadian rhythm Period2 [23,90]. Both β-TrCP1 and β-TrCP2 are biochemically indistinguishable in vitro and it is not clear if they recognize a unique set of substrates in vivo.

Mice deficient in β-TrCP1-are viable, with normal circadian rhythm and only minor defects in fertility. Animals do not exhibit any apparent defects up to 16 months of age [86,91,92,93]. Isolated mouse embryonic fibroblasts (MEFs) have a reduced growth rate, increased size, and abnormal ploidy. Upon Wnt3a stimulation, they show more stable nuclear β-catenin accumulation.

The β-TrCP-deficient male germ cells do not enter meiosis but instead undergo apoptosis. The early mitotic inhibitor 1 (Emi1) accumulation appears to contribute to the slight impairment in spermatogenesis and male fertility [23,92]. Another study shows that simultaneous inactivation of β-TrCP2 expression via inducible shRNA (which reduced β-TrCP2 to ∼10% of the original level) leads to more severe testicular defects in otherwise viable and healthy animals. Authors of the study were able to rescue the defect by β-TrCP2 restoration and attributed the observed defect to ineffective degradation of the Snail1 transcription factor [94]. This transcription factor is important for epithelial–mesenchymal transition and its degradation is necessary for the proper development of cell adhesion within the seminiferous tubules. Depletion of Snail1 completely rescues spermatogenesis in β-TrCP1-deficient mice. Another research group used testis-specific inactivation of β-TrCP2 in the context of β-TrCP1-deficient mice. The authors also observed spermatogenesis impairment but attributed this defect to inappropriate degradation of the Doublesex- and mab-3-related transcription factor 1 (Dmrt1) involved in the mitosis–meiosis transition in mouse male germ cells [91].

Ubiquitin ligase β-TrCP1 is also an important factor in other tissues’ homeostasis. Contrary to the epidermal or intestinal epithelium, mammary glands of β-TrCP1-deficient female mice display a hypoplastic phenotype [95]. A β-TrCP1-deficient retina shows a complete absence of cholinergic amacrine cells (CACs), decrease in tyrosine hydroxylase-expressing amacrine cells, and reduction in the number of retinal ganglion cells. The population of precursors of CACs is reduced, whereas the population of precursors of retinal ganglion cells increases [96]. The intestine-specific tamoxifen-inducible ablation of both β-TrCP1 and β-TrCP2 results in β-catenin and IκBα stabilization and leads to mucositis, a deleterious gut mucosal inflammation resulting in mucosal barrier defects. The increased NF-κB-independent production of interleukin 1β (IL-1β) is responsible for mucosal barrier defects, and inhibition of IL-1β partially rescues the inflammatory phenotype [97].

Contrary to the mild phenotype of β-TrCP1 deficiency, inactivation of β-TrCP2 results in the developmental arrest and the embryonic death before E10.5 [98]. Embryos lacking β-TrCP2 manifest accumulation of the cyclin-dependent kinase (CDK) inhibitor p19Arf in the yolk sac, but the concomitant inactivation of p19Arf does not rescue the lethal phenotype.

#### 3.1.2. Zinc and RING Finger 3/ RING Finger 43 (ZNRF3/RNF43)

Both ZNRF3/RNF43 are RING-type transmembrane ubiquitin ligases containing the protease-associated ectodomain in the extracellular part. As mentioned, they target the Fzd receptor and mediate its ubiquitination and degradation which leads to attenuation of Wnt signaling. Both RNF43 and ZNRF3 are mutated in pancreatic carcinomas and colorectal and endometrial cancer [99,100].

Mice deficient in Znrf3 die shortly after birth. They have impaired lens development and about 20% of embryos show neural tube closure defects [38]. Simultaneous deletion of both genes (*Znrf3* and *RNF43*) in the intestine leads to the epithelial hyperproliferation phenotype similar to one observed in Apc^min^ mice with constitutively active β-catenin [39]. This phenotype is dependent on paracrine delivery of Wnt3, and the simultaneous inactivation of the Wnt-secretion co-factor Porcupine abrogates the enhanced epithelial proliferation [101]. Expectedly, the adrenocortical deletion of *ZNRF3*, but not *RNF43* (which is not expressed at a significant level in the adrenal cortex), leads to a hyperproliferative phenotype as well. The ZNFR3-dependent expansion is restricted only to the inner zone of the adrenal cortex and does not phenocopy β-catenin hyperactivation [102]. A ZNRF3 deficiency also leads to a disrupted testis determination. This is in agreement with the observation that testis development depends on Wnt signaling inhibition [103]. Mice without Znrf3 have a gonadal reversal in E12.5 and it depends on ectopic Wnt signaling during sex determination [104].

#### 3.1.3. RING Finger 146 (RNF146)

Ubiquitin ligase RNF146 contains the N-terminal RING domain and the WWE domain. It mediates the ubiquitination of proteins PARylated by Tankyrase and its expression level was significantly elevated in a subset of non-small cell lung cancer and colorectal cancer [105].

Mice deficient in RNF146 die during embryogenesis. They are smaller with a delayed bone formation in the calvarium [55]. Mice with the osteoblast-specific RNF146 deletion die perinatally due to the fact of respiratory failure. Embryos have a short stature, fail to close fontanelles, exhibit hypomineralization of the calvarium, have small clavicles, and are osteopenic, with low serum levels of osteocalcin. The phenotype mimics some features observed in patients with cleidocranial dysplasia. Loss of RNF146 results in AXIN stabilization in osteoblasts and inhibition of the Wnt signaling pathway. Defective expression of the Wnt target fibroblasts growth factor 18 (FGF18) leads to inhibition of mitogen-activated protein kinase (MAPK) activity and, subsequently, to decreased osteoblast proliferation. As a consequence of reduced osteocalcin production, the osteoblasts-specific Rnf146-deficient mice exhibit an increase in bone marrow fat stores and glucose intolerance [55].

Contrary to its role in osteoblasts, Wnt signaling inhibits osteoclastogenesis [106]. A major osteoclast factor Rankl restricts Wnt activation via suppression of Rnf146 expression and Axin stabilization. Accordingly, macrophage-specific deletion of *Rnf146* triggers accelerated osteoclastogenesis [107]. Besides Axin, RNF146 is responsible for the degradation of the SH3 domain-binding protein 2 (SH3BP2). Stabilized SH3BP2 potentiates RANKL signaling and osteoclastogenesis, mimicking its “gain-of-function” mutations found in patients with hereditary cherubism [108,109].

#### 3.1.4. RING Finger 61 (RNF61)

Ubiquitin ligase RNF61 is a member of the putative RNA-binding protein family characterized by unusual C3H-Zinc finger domains and the RING domain. Telomerase reverse transcriptase (TERT), phosphatase and tensin homolog (PTEN), adenomatous polyposis coli (APC) or AMP-activated protein kinase (AMPK) are among its potential substrates.

Mice deficient in Rnf61 show chronic AMPK activation in both liver and adipose tissue, resulting in significant suppression of the diet-induced metabolic syndrome [88]. Although no clear connection exists between this phenotype and deregulated Wnt signaling, there is well-described crosstalk between AMPK and β-catenin activity [110,111,112]. Moreover, AMPK activation by metformin was recently shown to inhibit β-catenin stabilization [113]. Thus, Rnf61-dependent ubiquitination and degradation of AMPK and APC could represent an interesting feedback mechanism between Wnt signaling and metabolism activity.

#### 3.1.5. Seven in Absentia Homolog (SIAH)

Seven in Absentia Homolog 1/2 ubiquitin ligases are homologous proteins consisting of the N-terminal RING domain, two zinc-finger domains, and the substrate-binding domain. There is a number of substrates subjected to degradation mediated by SIAH1/2 ubiquitin ligases. Under hypoxic conditions, SIAH1/2 mediate ubiquitination and degradation of prolyl hydroxylase 1 (PHD1) and PHD3. As a consequence, the hypoxia-inducible factor 1-α (HIF1α) is stabilized and the hypoxia response transcription program is initiated [114].

Seven in Absentia Homolog 1a-deficient mice exhibit growth retardation or early lethality (about 70% of SIAH1a-deficient pups die during the nursing period). They are sterile with defective spermatogenesis due to the impairment of meiotic progression [115]. Mice deficient in Siah1a suffer from osteopenia [116]. Interestingly, Siah2-deficient mice are normal, healthy, and fertile. They have a significant expansion of myeloid progenitor cells and osteoclasts in the bone marrow [117]. Embryos lacking both *SIAH* genes die within several hours of birth. They do not have any obvious defects, and the cause of death remains to be determined. Notably, both Siah1 and Siah2 have been shown to play a role in hypoxia and unfolded protein response (UPR) and their absence can result in a complex phenotype caused by oxidative and proteotoxic cellular stress [117,118,119,120].

#### 3.1.6. E3 Isolated by Differential Display (EDD)

The EDD/UBR5 ubiquitin ligase belongs to the HECT ligase family. It has the UBA (ubiquitin-associated) domain in its N-terminus, a centrally located UBR-type zinc finger involved in the recognition of N-terminal degrons and the C-terminal HECT domain. E3 isolated by differential display is predominantly localized in the nucleus. The EDD was shown to target RNF168 ubiquitin ligase and TLE/Groucho repressors for proteasomal degradation [121]. It is deregulated in many types of cancer, e.g., breast and ovarian cancer or mantle cell carcinoma [122].

E3 isolated by differential display-deficient mice have significant developmental arrest characterized by defective vascular development in the yolk sac and allantois, along with defective chorioallantoic fusion [123]. The authors discussed that these extraembryonic defects presumably compromise fetal–maternal circulation, leading to a general failure of embryonic cell proliferation and widespread apoptosis. It is of note that mice deficient in Wnt receptor Fzd5 have similar defects in the yolk sac and placental vasculogenesis [124]. Nevertheless, there is no study of Wnt signaling deregulation in Edd-deficient mice. To investigate the EDD physiologic role in this pathway and generally in mouse development, it is necessary to prepare conditional mouse models. So far only limb bud-specific *Edd* deletion has been reported with no obvious morphological or developmental defects [125].

#### 3.1.7. Neural Precursor Cell Expressed, Developmentally Downregulated 4 (Nedd4)

The Nedd4 ubiquitin ligase belongs to the NEDD-type HECT ligase family. It has a characteristic structure common for all NEDD4 family members: the N-terminal C2 domain, four WW domains, and the C-terminal HECT domain. The NEDD4 was proposed to interact with the tumor suppressor PTEN and to mediate its ubiquitination. It has been also shown to play a role in the regulation of Epithelial Na^+^ channel (ENaC) and RNA polymerase 2. The NEDD4 is often overexpressed in many types of human malignancies, e.g., prostate, bladder, colorectal, gastric or breast carcinoma [126].

Mice heterozygous for Nedd4 are moderately insulin-resistant but protected against high-fat diet (HFD)-induced obesity. They show less body weight gain, less fat mass, and smaller adipocytes [127]. Knockout mice die perinatally [128,129,130]. They are growth-retarded around E11.5 with signs of subcutaneous bleeding in more than half of embryos. At E18.5, embryos do not display any spontaneous movement. The neural circuits are still active as the embryos react to mild pinches. Diaphragm muscles are significantly thinner and fragile with wavy and disorganized muscle fibers. Mice have an impaired innervation pattern in diaphragm muscles, leaving a gap at its ventral region. The Schwann cells differentiation is intact but they have a lower number of axons and motoneurons, impaired formation of neuromuscular synapses, and abnormal neuromuscular synaptic activity [128]. In another study, Nedd4-deficient neurons had more immature dendrites and showed significantly reduced apical dendrite branching, synaptic transmission, and synapse numbers. The authors revealed that the major substrate of Nedd4 involved in neuronal branching regulation is the Ras-related protein 2a (Rap2a) and that the expression of dominant-negative Rap2A rescues correct dendritogenesis [129]. Another research group presents that Nedd4-deficient mice show prominent heart defects (double-outlet right ventricle and atrioventricular cushion defects) and vasculature abnormalities [131]. Follow-up research pinpointed that the inhibitor of insulin signaling the growth factor receptor-bound protein 10 (Grb10) is a major substrate of the Nedd4 ubiquitin ligase. Its stabilization is responsible for delayed embryonic development, reduced growth, body weight, and neonatal lethality. Mechanistically, the stabilized Grb10 inhibits the insulin-like growth factor 1 receptor (Igf1R) cell surface localization. The Grb10 heterozygosity rescues the Nedd4 deficiency lethal phenotype [130]. Vascular-specific deletion of *Nedd4* displays deformed aortas with disarranged elastin fibers. It also results in increased vascular calcification and bone-related marker expression in aortas [132]. The bone-specific Nedd4-mutant mice show enhanced bone mass accrual and upregulated gene expression of osteogenic markers in the bone. Bone formation is decreased, and the proliferation of primary osteoblasts isolated from calvaria is higher. The number and surface area of tibial osteoblasts are higher as well [133]. The neural crest cell-specific Nedd4 deficiency results in significant craniofacial defects with reduction of a cranial bone and decrease in osteoblasts numbers. The Nedd4 seems to be essential for neural crest stem cell self-renewal and survival [134,135]. T cells from Nedd4-deficient fetal liver chimeras display a naïve T cell phenotype. T cells develop normally but proliferate less and their ability to activate B cells is diminished. Biochemically, upon CD28 co-stimulation, Nedd4 controls the stability of another ubiquitin ligase Cbl-b. Inappropriately stabilized Cbl-b ligase targets the T cell receptor (TCR) and its components and ultimately blocks T cell activation and function [136]. Interestingly, Nedd4 deficiency abrogates T cell hyperactivity in Cbl-b-deficient mice [137]. Animals with intestine-specific deletion of *Nedd4* when crossed with APC^+/min^ mice have enhanced tumor growth and Wnt signaling [89].

#### 3.1.8. Neural Precursor Cell Expressed, Developmentally Downregulated 4-Like (NEDD4L)

The NEDD4L ubiquitin ligase is a close homolog to NEDD4 and they share a common structure. The NEDD4L regulates numerous ion channels, especially ENaC [29]. As mentioned in Section 4.1., NEDD4L also mediates degradation of phosphorylated Smad2 and Smad3, and associates with TGF-βRI via the Smad7 adaptor leading to destabilization of the receptor. The polymorphism causing premature truncation of the NEDD4L protein is associated with essential hypertension [138].

Neural precursor cell expressed, developmentally downregulated 4-like heterozygous mice are viable but hyperactive [139]. The mouse model of NEDD4L with complete deficiency suggests that the Nedd4L major ubiquitination target is ENaC [140,141]. This sodium channel is involved in the reabsorption of sodium ions in the kidney, colon, and lungs [142]. It is also necessary for the saltiness perception associated with taste buds [143]. Neural precursor cell expressed, developmentally downregulated 4-like binds to the PY motifs of ENaC subunits via its WW domains. This interaction is responsible for ENaC ubiquitination and subsequent downregulation on the apical membrane. There are two independent mouse models of Nedd4L deficiency. The possibly hypomorphic Nedd4L knockout model has a relatively mild phenotype with higher blood pressure in both normal and high-salt diets. Concurrent administration of the ENaC inhibitor amiloride rescues the hypertension phenotype. Moreover, a chronic high-salt diet leads to cardiac hypertrophy [144]. In the second model of Nedd4L deficiency, the embryos have collapsed alveolar spaces and die perinatally [145]. The kidney-specific knockout mice suffer from a progressive kidney injury phenotype associated with increased sodium ion reabsorption, hypertension, and reduced levels of aldosterone. The phenotype is manifested by fibrosis, higher apoptosis, and cystic tubules [146]. In the mast cell-specific knockout, NEDD4L limits the intensity and duration of immunoglobulin E (IgE)-Fc_ε_RI-induced positive signal transduction. It appears that in mast cells, the tyrosine kinase Syk is the main substrate for the NEDD4L ligase [147].

#### 3.1.9. ITCH

The ITCH ubiquitin ligase belongs to the NEDD-type HECT ligase family. It contains the N-terminal C2 domain, four WW domains, and the HECT domain. Ubiquitin ligase ITCH regulates the stability of transmembrane receptors through monoubiquitination and intracellular proteins through polyubiquitination. It drives the monoubiquitinated and polyubiquitinated substrates to lysosomal and proteasomal degradation, respectively [148]. Reportedly, for proliferation- and survival-associated proteins c-Jun, JunB, p63, Notch, and glioma-associated oncogene homolog 1 (GLI1), TGF-β activated kinase 1 binding protein 1 (TAB1) belong to their substrates [149,150]. Mutations in ITCH cause inflammation, including inflammatory bowel disease or nephritis, and the ITCH deficiency is associated with multisystem autoimmune disease [151].

The non-agouti-lethal *Itchy* mice suffer from severe immune and inflammatory defects which result in persistent scratching of the skin [152,153]. On the C57BL/10 background, Itch deficiency is associated with the spontaneous development of a late-onset and progressively lethal systemic autoimmune-like disease, characterized by lymphoproliferation in the spleen, lymph nodes, and medulla of the thymus and by chronic pulmonary interstitial inflammation. The usual cause of death of these animals is hypoxia. On the JU/Ct background, *Itchy* mice develop an inflammatory disease of the large intestine [153].

For *Itchy* mice, T cells proliferate and adopt an activated phenotype. Production of the Th2 (T helper cell type 2) cytokines IL-4 and IL-5 is augmented upon stimulation, and the Th2-dependent serum concentrations of IgG1 and IgE are increased [154]. The phenotype is partially caused by the dysregulation of regulatory T (Treg) cells in the absence of the ITCH ligase. Treg cell-specific ablation of the Itch E3 ubiquitin ligase causes massive multiorgan lymphocyte infiltration and skin lesions, chronic Th2 cell activation, and the development of severe antigen-induced airway inflammation. The Itch-deficient Treg cells express a higher amount of Th2 cytokines and they are able to instruct naïve T cells to differentiate into Th2 effector cells [155]. The follow-up research has shown that Itch is essential for the differentiation of follicular B helper T cells (T_FH_), germinal center response, and IgG production following acute viral infection. The development of T_FH_ cells is halted in early stages, and Itch acts intrinsically in CD4+ T cells. At the molecular level, during T_FH_ cell development, the Itch ubiquitin ligase controls the stability of the transcription factor Foxo1 [156]. Mice deficient in the E3 ubiquitin ligases CBL-b and Itch show an increase in T cell activation and display spontaneous autoimmunity. The double-mutant T cells show increased phosphorylation of the TCR-ζ chain, but TCR complex stability and membrane location are intact [157].

Keratinocyte-specific knockout of ITCH revealed its contribution to skin development and wound healing which is independent of the immunological phenotype observed in *Itchy* mice [158]. Moreover, *Itchy* females have reduced implantation sites, decreased corpora lutea, and increased estrous cycle length [159]. Mice deficient in Itch fed a HFD do not gain weight and do not show insulin resistance. It seems that Itch deficiency protects mice from obesity-related non-alcoholic fatty liver disease. Deficient animals have an accelerated metabolism and higher expression of genes involved in fatty acid oxidation. As a result of aberrant T helper cells activation, mutant mice exhibit a lower amount of M2 (obese adipose tissue)-type macrophages [160]. Moreover, Itch deficiency renders mice resistant to tumor necrosis factor-α (TNF-α)-induced acute liver failure in three distinct models [161].

The E3 ubiquitin ligase Itch negatively regulates the development and function of hematopoietic stem cells (HSCs). Specifically, HSCs deficient in Itch are more competent, have longer repopulating activity, accelerated proliferation rates, and sustained progenitor properties. They also display an accumulation of the activated Notch1 receptor. Consistently, knockdown of Notch1 in Itch-deficient HSCs results in reversion of the phenotype [162].

#### 3.1.10. Casitas B-Lineage Lymphoma (CBL)

Both c-CBL and CBL-b are close homologs from the CBL family and share the N-terminal tyrosine-kinase-binding domain, a linker, and the RING domain [163,164]. Expression of c-CBL is broad with the highest level in the thymus and testes. In activated T cells, it is a prominent target of tyrosine kinases [165].

Mice deficient for either c-CBL or CBL-b show T cell hyperactivation, which is driven by lowering the TCR affinity/avidity threshold and loss of the co-receptor signal requirement [166,167,168,169]. While c-Cbl-deficient mice have hyperactive thymocytes, CBL-b-deficient mice have activated mature T cells. Double-positive (CD4+CD8+) thymocytes lacking c-CBL display a higher amount of the membrane-bound TCR/CD3 complex—CD4 and CD8 receptors and tyrosine kinases Lck and Fyn [166]. An elevated level of the TCR complex can be a result of TCR ζ-chain stabilization [170,171]. Mice deficient in Cbl-b have a normal thymus and thymocyte development, but they display hyperproliferation of peripheral T cells. Similarly to CBL-b deficiency, there is no requirement for the second signal, and sole CD3 stimulation leads to T cell activation and proliferation. As a result of inadequate control of T cell activation, the CBL-b mouse model is susceptible to experimentally induced or spontaneous autoimmune diseases such as arthritis and diabetes [172]. Effector T cells in these animals are also insensitive to Treg on the cellular level mediated suppression of its mediator TGF-β [173].

As mentioned above, the c-CBL-deficient mice phenotype is mainly associated with thymocytes hyperactivation and proliferation. On the biochemical level, they display increased protein activation of the tyrosine kinase Lck and Zap-70 and the downstream effectors linker for activation of T cells (Lat) and SH2 domain containing leukocyte protein of 76 kDa (Slp-76). Protein kinase Zap-70 is able to mediate c-CBL interaction with TCRζ but it is probably not the direct target of its ubiquitin ligase activity [170]. Based on the results from c-CBL-deficient thymocytes, it seems that c-CBL can regulate different stages of T cell development, maturation, and selection processes.

The mammary fat pads of c-CBL–mutant female mice show increased ductal density and branching [167]. The decrease in motility of c-CBL-deficient osteoclasts results in a decreased ability of osteoclasts to invade and resorb bone and mineralized cartilage in vivo [174]. Mice deficient in c-Cbl exhibit an increase in whole-body energy expenditure, decrease in adiposity, and an increase in food intake, reduced circulating insulin, leptin, and triglyceride levels and improved glucose tolerance [175]. These changes are accompanied by a significant increase in mouse activity (2 to 3 fold).

Both c-CBL and CBL-b double knockout (DKO) mice are embryonically lethal before E10.5, which suggests that these ligases have important overlapping functions in embryonic development. T cell-specific DKO leads to an exaggeration of the immune phenotype as T cells become hyperresponsive upon CD3 stimulation [168]. The DKO T cells do not downregulate surface TCR after antibody engagement, which results in continuous TCR signaling [168]. The germinal center B cells deficient in both ligases display an early exit of high-affinity antigen-specific B cells from the germinal center reaction and, therefore, impaired clonal expansion [176]. The mouse model of mast cell-specific deficiency of both ligases shows that CBL-b, but not c-CBL, functions as a negative regulator of FcεRI-induced degranulation [177]. The mouse DKO model in HSCs develops a myeloproliferative disorder. The HSCs of c-CBL-deficient mice exhibit only an augmented pool size, hyperproliferation, greater competence, and enhanced long-term repopulating capacity [178]. The mammary gland-specific DKO shows CBL-b and c-CBL redundant function in mammary stem cell renewal [179].

## 4. TGF-β Signaling Pathway

**Figure 4:**
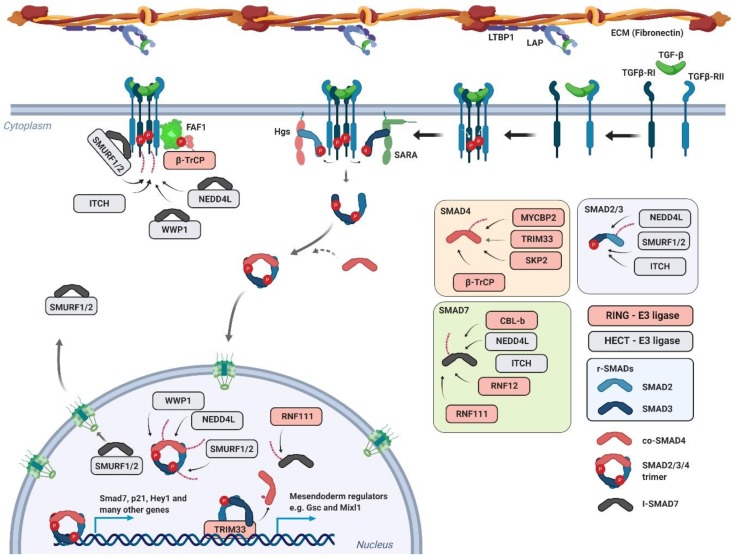
The TGF-β signaling pathway and its regulation by ubiqutination TGF-β released from the LTBP1 complex binds to TGF-β receptor II (TGF-βRII) and initiates transphosphorylation and activation of the associated TGF-β receptor I (TGF-βRI). The fully activated receptor transduces the signal to downstream factors belonging to the family of r-Smad (regulatory-Small mothers against decapentaplegic) transcription factors—Smad2 and Smad3. Smad2/3 form a trimer complex with Smad4 (co-Smad), translocate to the nucleus, and initiate transcription of TGF-β target genes. For details see the text.

Transforming growth factor-β belongs to a distinct family of extracellular soluble protein ligands involved in diverse developmental and homeostatic processes of higher eukaryotes [180]. During maturation, TGF-β homodimer forms a complex with a LAP (latency-associated peptide) originally derived from the region of the TGF-β protein between the signaling peptide and the C-terminally located active TGF-β ligand. This small latent complex (SLC) associates with latent TGF-β-binding protein 1 (LTBP1) and it is sequestrated to the fibronectin-based extracellular matrix [181]. Following proteolytic activation, the released TGF-β binds to the TGF-β receptor II (TGF-βRII) and initiates transphosphorylation of the associated TGF-β receptor I (TGF-βRI) (Figure 4) [182]. These events lead to full activation of the serine/threonine kinase located in the intracellular part of TGF-βRI. The activated receptor transduces the signal to downstream factors belonging to the family of regulatory-Smad (r-Smad) transcription factors—Smad2 and Smad3. Phosphorylation of these factors depends on the FYVE (Fab1p, YOTB, Vac1p and EEA1) domain containing protein Smad anchor for receptor activation (SARA) or hepatocyte growth-factor-regulated tyrosine kinase substrate (HGS) [183,184]. These membrane-associated proteins are responsible for delivering Smad factors to the vicinity of the activated receptor complex. Once phosphorylated, Smad2/3 form a trimer complex with Smad4 (co-Smad4) and translocate to the nucleus. The Smads mad homology 1 (MH1) domain is responsible for the specific association with target gene promoters, whereas the mad homology 2 (MH2) domain is responsible for interactions with transcriptional co-factors, transactivators, and other regulators [185,186]. The fully assembled complex initiates expression of TGF-β target genes and, ultimately, activates transcriptional programs which govern and execute tasks like cell cycle inhibition or transdifferentiation [187,188].

### 4.1. TGF-β Signaling Pathway and its Regulation by Ubiquitin Ligases

The Smad homolog *Smad7* is one of the early target genes of TGF-β signaling [189]. It lacks the N-terminally located MH1 domain but still contains the receptor-binding MH2 domain. In the linker region between N- and C-terminal parts, Smad7 contains the PPxY motif which is responsible for E3 ubiquitin ligase SMURF2 (Smad ubiquitination regulatory factor 2) association [190,191].

Ubiquitin ligase SMURF2 associates with Smad7 in the nucleus and their complex subsequently translocates to the cytosol. The complex then interacts with the activated receptor and causes its proteasome-dependent degradation, which ultimately leads to the inhibition or attenuation of TGF-β signaling. During the TGF-β stimulated epithelial–mesenchymal transition (EMT), SMURF-dependent ubiquitination and degradation of TGF-βRI is blocked by concurrent action of another ubiquitin ligase TNF receptor-associated factor 4 (TRAF4), a member of the RING domain containing E3 ubiquitin ligase family [192,193,194]. Transforming growth factor β receptor-associated TRAF4 potentiates TGF-β signaling by mediating ubiquitination and proteasome degradation of SMURF. At the same time, it was shown that TRAF4 is also responsible for signaling-type Lys63-linked ubiquitination of TGF-βR, leading to its association with TGF-β activated kinase 1 (TAK1). Ubiquitin ligase SMURF2 also interacts with Smad2/3. It mediates Smad2 ubiquitination and targets it for degradation [195]. Its activity towards Smad3 is much weaker and leads to multiple monoubiquitinations of Smad3. This seems to have no effect on Smad3 stability, but rather on its ability to form a complex with Smad4 [196]. Interestingly, SMURF2 also targets Smad inhibitors Sloan–Kettering Institute (SKI) proteins. It binds them indirectly using Smad2 as an adapter [197].

Another E3 ubiquitin ligase that has been shown to effectively regulate the canonical TGF-β pathway is NEDD4L (NEDD4-like). The activated Smad complex is phosphorylated in the nucleus in a series of events initiated by CDK8/9 [198]. These transcription-associated kinases prime the Smad complex to another round of phosphorylation via GSK3β. The GSK3β-phosphorylated motif is recognized by the WW domain of the HECT-type ubiquitin ligase NEDD4L [199]. NEDD4L-dependent ubiquitination leads to the proteasome-dependent degradation of Smad2/3 and attenuation of TGF-β signaling. Interestingly, the WW domain is flanked by two serines targeted by serum/glucocorticoid regulated kinase 1 (SGK1). This phosphorylation inhibits NEDD4L interaction with Smads and promotes their stability. The ubiquitin ligase NEDD4L and another HECT-type ligase WW domain containing E3 ubiquitin protein ligase 1 (WWP1) were also shown to recognize TGF-βRI in a similar manner as SMURF (Smad7–dependent). The receptor ubiquitination decreases its stability on the membrane and leads to its internalization and subsequent degradation.

Additionally, the HECT-type ITCH ligase positively regulates TGF-β signaling as well. It binds Smad2 but does not have any effect on its stability. It rather promotes, in a HECT-dependent manner, its association with TGF-βR [200]. A possible explanation can be drawn from other studies which have shown that ITCH promotes TGF-β signaling by mediating TGF-βR-dependent degradation of Smad-signaling inhibitors, Smad7 and Ras association domain family 1 isoform A (RASSF1A) [201,202].

The Smad7 protein is also a substrate for ubiquitin ligases RNF111 (Arkadia) and RNF12 (RLIM), which both localize to the nucleus and most probably target Smad7 in this compartment [203,204,205,206]. Importantly, RNF111 has a chain of Small ubiquitin-like modifier (SUMO)-interacting motifs in its N-terminal part, and it is possible that it recognizes its substrates once they are sumoylated. Moreover, it has displayed activity towards Smad transcriptional co-repressors SKIL and SKI [207,208,209]. The protein involved in β-catenin degradation, AXIN, was shown to be a scaffold protein linking the RNF111 ubiquitin ligase and Smad7 [205]. It seems that Rnf12 can recognize Smad7 in the context of its interaction with SMURF [210]. In T cells, CBL-b is another RING ligase targeting Smad7. CBL-b and Smad7 interact physically and genetically, as it was shown that Smad7 inactivation restores the TGF-β signaling defect in CBL-b-deficient T cells [211].

Cullin1-dependent ubiquitin ligase β-TrCP was shown to mediate degradation of TGF-βR. The β-TrCP protein does not bind to the receptor directly but via the linker protein FAS-associated factor 1 (FAF1) [212]. Upon phosphorylation by the AKT kinase, FAF1 relocates to the plasma membrane where it interacts with TGF-βR and mediates its ubiquitination via the β-TrCP-ubiquitin ligase. This represents interesting crosstalk between PI3-K and TGF-β signaling.

Additionally, the E3 ubiquitin ligase TRIM33 (TIF-1γ or ectodermin) was also shown to interact with the Smad2/3 complex [213,214]. There are conflicting studies regarding its role in TGF-β signaling. It was proposed that TRIM33 and Smad4 associate with the r-Smads in a mutually exclusive manner [215]. Another study suggests that TRIM33 is a bona fide ubiquitin ligase for Smad4 which mediates chromatin-associated Smad4 monoubiquitination [216].

Last two ubiquitin ligases involved in TGF-β signaling are MYC binding protein 2 (MYCBP2) and S-phase kinase associated protein 2 (SKP2) [217,218,219]. Putative RING finger E3 ubiquitin ligase MYCBP2 could regulate Smad stability in neurons. *Drosophila* MYCBP2 homolog Highwire (Hiw) interacts with the *Drosophila* Smad homolog Medea. Highwire-mutant flies have also unrestrained synaptic growth [218]. Biochemically, Hiw controls the level of *Drosophila* TGF-βR (Tkv) and this could be involved in restricting Medea signalization in *Drosophila* neurons and intestinal stem cells [217]. The cullin1 ubiquitin ligase-dependent substrate adapter SKP2 mediates degradation of Smad4. It preferentially binds cancer-associated forms of Smad4 [219].

### 4.2. Mouse Models of Ubiquitin Ligases Involved in the TGF-β Signaling Pathway

Since the TGF-β signalling pathway has a crucial role in diverse developmental processes, mouse models deficient in its core components exhibit a wide range of developmental defects [220,221]. As mentioned above, several ubiquitin ligases have been suggested to regulate the TGF-β signalling pathway. However, mouse models of their deficiency show that these ubiquitin ligases often have different functions. While phenotypes of RNF111 (Arkadia) and TRIM33-deficient mice supported a crucial role in the TGF-β signaling pathway, the mouse models examining the deficiencies of other ubiquitin ligases failed to bring evidence of involvement in TGF-β signaling [214,222]. They seem to have other physiological substrates instead and, thus, control different processes as discussed in more detail below.

#### 4.2.1. Smad Ubiquitination Regulatory Factor (SMURF)

Ubiquitin ligases SMURF1/2 share a high protein sequence homology (>70%) and their domain architecture is similar. Structurally they belong to NEDD-type HECT ligases. The N-terminal protein kinase C (PKC)-related C2 domain is followed by two or three WW PPxY/substrate interacting domains, respectively, and the catalytical C-terminal HECT domain. As mentioned above, SMURF1–2 were implicated in activated Smad2/3 and TGF-βRI degradation. Intracellular localization of SMURF1/2 is ambiguous as they were found in both the cytosol and the nucleus. Overexpression of SMURF1/2 was found in many cancer tissues and is associated with worse patient survival.

Individual SMURF1- and SMURF2-deficient mice are viable and fertile without any noticeable defects in embryogenesis [223,224]. The SMURF1 absence leads to an age-dependent bone mass increase due to the enhanced osteoblast activity [223]. One line of evidence shows that SMURF1 inhibits mesenchymal stem cell (MSC) differentiation and their osteoblastic potential via controlling the stability of the transcriptional factor JunB. Simultaneous inactivation of JunB in vitro rescued the osteogenic potential of MSCs to the normal level [225]. Another group revealed that the osteoblast Smurf1 ubiquitin ligase activity was directed towards another factor, Map kinase kinase 2 (Mekk2), an upstream kinase in the Jnk signaling cascade. Hyperactive Jnk signalization was then responsible for the higher activity of osteoblasts [223]. It was also reported that bone loss observed in mice with artificially increased TNF-α signaling was dependent on SMURF1 activation [226]. Another age-dependent phenotype of SMURF1-deficient mice was the spontaneous development of hepatic steatosis. In hepatocytes, the SMURF1 deficiency leads to upregulation of the expression of peroxisome proliferator-activated receptor γ (PPARγ) and its target genes involved in lipid synthesis and fatty acid uptake. In this context, however, SMURF1 does not mediate protein degradation of PPARγ but rather inhibited its activity via the non-proteolytic K63-linked ubiquitin modification. Treatment of SMURF1-deficient mice with a PPARγ antagonist, GW9662, reversed the lipid accumulation in the mutant mice liver [227].

The SMURF2-deficient mice display an expanded HSC compartment in the bone marrow with a higher repopulating capacity especially in aged animals [224]. The Smurf2 deficiency renders mice susceptible to spontaneous tumorigenesis, most notably the B-cell lymphoma, which resembles human diffuse large B-cell (DLBC) lymphoma with molecular features of germinal or post-germinal center B cells [228]. Mechanistically, Smurf2 is responsible for the degradation of Yin Yang 1 (YY1), a key germinal center transcription factor. Stabilized YY1 is responsible for transactivation of the c-Myc oncogene and activation of B cell proliferation [229].

Transgenic mice deficient for both SMURF1 and SMURF2 display complex developmental defects. Approximately one-third of mutant embryos display gastrulation defects characterized by abnormal posterior structures. The rest of the embryos gastrulate normally but show gross developmental abnormalities including an open neural tube and lateral expansion of the neuroectoderm [87]. This is a feature characteristic for mice with defects in planar cell polarity (PCP) and convergent extension movements (CE). Indeed, mice with only one of the four *SMURFS* alleles (e.g., *SMURF1*^−/+^; *SMURF2*^−/−^) show stereocilia misalignment on the cochlear organ of Corti. Mechanistically, SMURF deficiency is responsible for inappropriate activation of the non-canonical Wnt signaling pathway. Wnt engagement to FZD receptor leads to Dishevelled 2 protein phosphorylation and its translocation to the membrane. The phosphorylated Dishevelled 2 binds the Prickle protein, a major regulator of PCP. This interaction is mediated by the Dishevelled 2 constitutive partner PAR6. The whole complex is later recognized by SMURF which is responsible for the ubiquitination and degradation of Prickle. Inappropriate Prickle degradation leads to PCP and CE defects in SMURF1/2 DKO mice [87].

#### 4.2.2. RING Finger 111/Arkadia

As mentioned above, RNF111 contains several SUMO-binding motifs and it can possibly recognize sumoylated substrates [230]. Mutations affecting RNF111 function were documented in patients with ovarian and colorectal cancer [231].

In mice, the *Arkadia* recessive mutation was generated using gene-trap mutagenesis. Heterozygous *Arkadia* mice are normal and healthy, yet they have reduced expression of several TGF-β target genes [231]. Developmental abnormalities of homozygous animals appear early in mouse embryogenesis. An antero-visceral endoderm (AVE) is formed but the embryo lacks a node, has a reduced head, fails to undergo turning, and dies very early at midgestation [232]. Similarly to Trim33-deficient mice, *Arkadia* mice exhibit defects associated with deregulated Nodal signaling. Embryonic cells show an accumulation of phosphorylated Smad2/3 proteins, yet surprisingly most of Nodal/Smad target genes are downregulated. As a result, *Arkadia* mice have a very similar phenotype-like Smad2-deficient embryos [222].

#### 4.2.3. S-Phase Kinase-Associated Protein 2 (SKP2)

S-phase kinase-associated protein 2 (SKP2) is the F-box protein which is characterized by five leucine-rich repeats in its N-terminus. S-phase kinase-associated protein 2 controls the stability of the CDK inhibitor p27 during the G1/S-phase transition [233]. The p27 protein is specifically recognized upon threonine phosphorylation and in the context of its complex with adapter proteins CDK regulatory subunit 1 (CKS1), CDK2, and cyclin E [234,235].

S-phase kinase-associated protein 2 knockout mice are small but viable [236]. Their cells have enlarged nuclei with polyploidy, aberrant centrosomes, and accumulated p27 protein. Simultaneous inactivation of the *CDKN1B*/*p27* gene reverts the phenotype [237]. When subjected to partial hepatectomy, SKP2-deficient mice exhibit proliferation-independent liver regeneration (via cellular enlargement) [238]. Similarly, scraping of the corneal epithelium in SKP2-deficient mice leads to defected wound healing [239]. Again, the phenotype was reversed by concurrent deletion of the *CDKN1B*/*p27* gene.

#### 4.2.4. MYC Binding Protein 2 (MYCBP2)

The MYCBP2 is a putative atypical RING ligase. It is a large protein containing the RCC1-like GEF domain, two PHR-family-specific domains, the RAE1-binding domain, the F-box binding domain 1, the Myc binding domain, and the C-terminal RING domain. Expression of MYCBP2 was found to be reduced in patients with acute lymphoblastic leukemia [240].

In mice, the genetic screen and targeted inactivation revealed MYCBP2 function in motor neuron pathfinding. In *Magellan*-mutant embryos, with the mutation causing MYCBP2 protein truncation, motor axons display navigation defects [241]. Surprisingly, they respond to guidance cues with normal sensitivity in vitro. Motor and sensory neurons from *Magellan* mutants show abnormal axon and growth cone morphologies. The phenotype is probably caused due to the disruption of the polarized distribution of the dual leucine zipper kinase (DLK), which acts upstream from p38Mapk and regulates microtubule stability. In accordance, the *Magellan* phenotype could be reversed by stabilizing microtubules with taxol or inhibiting p38Mapk activity. In a parallel study, the targeted conditional mutant shows that, as in invertebrates, MYCBP2 function is essential for shaping motor neurons terminals and the formation of major CNS axon tracts including those of the internal capsule. Major CNS axon tract phenotypes are partially caused by cell-non-autonomous mechanisms in a Dlk-independent manner [242]. The discrepancies among these models could be a result of different genomic deletions in the *MYCBP2* gene locus. In comparison to the *Magellan* mutant with the C-terminally truncated protein, the motor neuron-specific knockout mice have only 70 amino acid region proximal to the RCC1 domain deleted [241,242]. A follow-up study took advantage of these two models and prepared their cross [243]. The study focused on the previous observation that the MYCBP2 regulates mTOR signaling [244]. In agreement with this observation, mTOR signaling is attenuated in both models but, surprisingly, there are no mTOR signaling alterations in the prepared cross. This suggests that Mycbp2 regulates mTOR signaling via two independent pathways. Moreover, defective mTOR signaling is responsible only for certain neurodevelopmental defects (corpus callosum) associated with MYCBP2 deficiency but does not rescue the whole phenotype (defects in axon fiber tracts of the internal capsule and anterior commissure). Additionally, another study revealed that loss of MYCBP2 results in prolonged survival of severed axons in both the peripheral and central nervous systems. Survival of these axons depends on stabilization of mononucleotide adenyltransferase 2 [245].

#### 4.2.5. Tripartite Motif Containing 33 (TRIM33)

Tripartite motif containing 33 ubiquitin ligase is a member of the tripartite motif (TRIM) protein family. In its N-terminus, it has the RING finger, two B-box domains, and the coiled-coil domain. The C-terminal part of this protein is composed of the plant homeodomain (PHD) followed by the bromodomain. Tripartite motif containing 33 was found to be mutated or downregulated in several human cancers, like chronic myelomonocytic leukemia [246].

Mouse embryos deficient in Trim33 die at E9 [214,247]. They display a dramatic developmental delay. At E8.0–8.5 (3–6 somite pairs in controls), the TRIM33 mutant embryos are aligned at the base of the yolk sac, and, although they have formed the anterior-posterior body axis and recognizable head folds, it is difficult to identify any other embryonal structures. They are smaller and lack a clear distinction between epiblast and extra-embryonic ectoderm. Morphological and histological analyses demonstrated that TRIM33 mutants display remarkable defects in embryonic polarity and tissue patterning. The phenotype of mutant embryos was opposite to those observed in Nodal, Smad2, and Smad4 knockouts. Nodal induces and patterns the anterior visceral endoderm, and sustains trophoblast development. In TRIM33-deficient embryos, Nodal signaling becomes unrestricted leading to dramatic changes in the embryo body plan. In the epiblast, TRIM33 deficiency shifts mesoderm fates towards node/organizer fates. As a result of impaired primitive streak development embryos have defective mesoderm formation.

Mice with TRIM33 inactivation in monocytes, bone-marrow-derived macrophages, peritoneal macrophages, and neutrophils are normal and healthy. They show sustained expression of interferon-β1 at late stages of toll-like receptor-mediated activation in macrophages [248]. Hematopoietic stem cells’ specific loss of TRIM33 resulted in significant changes in erythroid, B-lymphoid, and myeloid compartments and decreased HSC capacity in transplantation assays [249]. The following study shows that a block in erythroid maturation in bone marrow was compensated with enhanced spleen erythropoiesis [250]. Another study on TRIM33 deficiency in HSCs showed that four-month-old mutant mice develop an accelerated aging phenotype in HSCs. The authors suggested that TRIM33 inhibition of TGF-β signaling was important for the balance between lymphoid and myeloid lineage differentiation and that myeloid- and lymphoid-biased HSC populations respond differently to TGF-β signaling. As the TGF-β signaling pathway was involved in the inhibition of HSC reentry into the cell cycle, disturbance in this signaling in TRIM33-mutant HSCs could result in the aging phenotype [251]. Tripartite motif containing 33-null mammary gland development appeared to be normal with no obvious developmental defects during the lifespan of virgin mice. However, after giving birth, the mice developed a significant lactation defect. Therefore, TRIM33 is probably essential for the terminal differentiation of alveolar epithelial cells in the mammary gland at the end of pregnancy.

#### 4.2.6. RING Finger 12 (RNF12)

Ubuquitin ligase RNF12 has the C-terminally located RING domain and the central basic domain (BD). It is involved in X-chromosome inactivation (XCI) and in mediating ubiquitination and degradation of pluripotency marker reduced expression protein 1 (REX1) [252,253]. Mutations in RNF12 were reported in X-linked intellectual disability.

Mice deficient in RNF12 mice display early embryonic lethality specific for female embryos due to the defectively imprinted XCI, precluding the development of embryonic trophoblast tissues. Males carrying a germline knockout of *RNF12* (Δ/Y) appear healthy and are fertile [254]. There is a study showing that only 50 percent of male neonates survive and those that die are significantly smaller, with altered lung branching and maturation [255]. Importantly, RNF12 is essential for triggering imprinted XCI but dispensable for random XCI [256]. Its crucial role is to maintain high Xist RNA levels, Xist clouds, and X-silencing in female embryos at blastocyst stages [257]. Mammary gland-specific knockout of RNF12 shows its requirement for alveolar morphogenesis and milk production. It acts as a survival factor for milk-producing alveolar cells. While mammary glands of virgin females contain many living RNF12-negative epithelial cells, lactating glands are only RNF12-positive. Moreover, decreased expression of RNF12 correlates with mammary gland involution [258].

#### 4.2.7. WW Domain Containing E3 Ubiquitin Protein Ligase (WWP)

Ubiquitin ligases WWP1/2 belong to the NEDD-type HECT ligase family [259]. They contain the N-terminal C2 domain, four WW domains, and the C-terminal HECT domain. They are both cytosolic and nuclear. Potential WWP1 substrates include TGF-βRI, Smad2 or Erb-B2 receptor tyrosine kinase 4 (ERBB4). Moreover, WWP2 was shown to mediate degradation of PTEN or the transcription factor OCT4. Interestingly, WWP1 is overexpressed in many types of cancer, especially prostate, breast, and liver, whereas WWP2 is frequently overexpressed in oral cancer.

Mice deficient in Wwp1 are viable and fertile without any obvious abnormalities. Embryos are born at the normal Mendelian ratio and grow relatively healthily. They develop increased bone mass as they age. This phenotype is associated with increased bone formation rates and normal bone resorption parameters [260,261]. They develop malformations of the craniofacial region. At the molecular level, Wwp2 is associated with Goosecoid, a transcriptional activator of the key cartilage regulatory protein Sox6. Importantly, WWP2 facilitates Goosecoid monoubiquitination, a post-translational modification required for its optimal transcriptional activity [260]. Mice defiecent in WWP2 also have reduced body and organ size and they resemble PTEN transgenic mice (Super-PTEN). In support of this, they have elevated and stabilized PTEN protein levels and reduced phosphorylation of the AKT kinase [262]. The bone marrow-derived macrophages have a stronger response to poly(I:C) challenge (regarding secreted TNF-α and IL-6 cytokines) and are more susceptible to poly(I:C)-induced death. These findings suggest that WWP2 negatively regulates TLR3-mediated innate immune and inflammatory responses. Indeed, WWP2 was shown to target adapter protein in TLR3-mediated NF-κB and IRF3 activation pathways (TRIF) for ubiquitination and degradation [261].

Mice deficient in both WWP1 and WWP2 display defects in axon–dendrite polarity in pyramidal neurons and abnormal laminar cortical distribution [263]. Interestingly, knockout of *miR-140*, encoded in *WWP2* intron, displayed similar phenotypic changes as those upon *WWP1* and *WWP2* deletion. The authors of the study delineated a novel regulatory pathway that involves the Sox9 transcription factor as a major regulator of WWP1/WWP2/miR-140 locus expression, and consequentially, axon specification, acquisition of pyramidal morphology, and accurate laminar distribution of cortical neurons.

## 5. Notch Signaling Pathway

**Figure 5:**
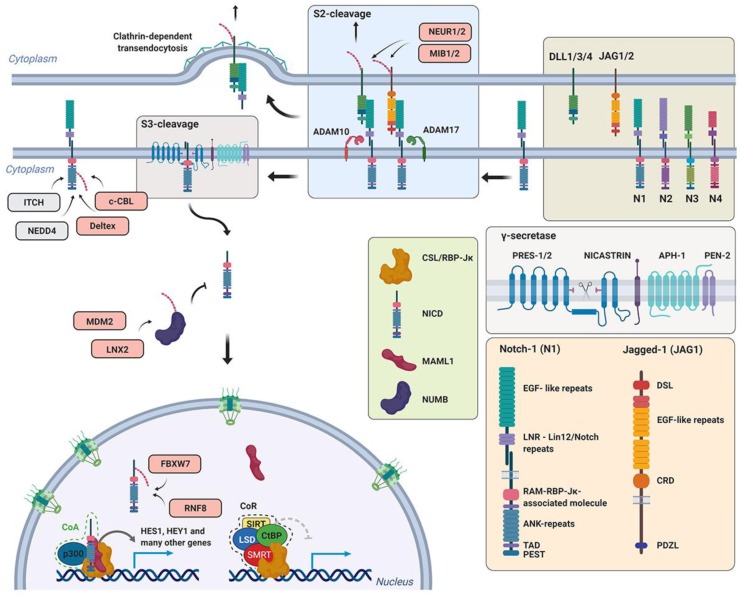
Notch signaling pathway by ubiqutination upon binding of Jagged/Delta (JAG/DLL) ligands to the Notch receptor (N1); the endocytic system is activated in the ligand-bearing cell. This leads to two sequential proteolytic events. The first is metalloproteinase-dependent (ADAM10/17) and it releases the extracellular domain of the Notch receptor which is afterward engulfed via transendocytosis. The second proteolytic cleavage is membrane bound and dependent on γ-secretase activity. After this step, NICD (the Notch intracellular part) is released to the cytosol and transported to the nucleus where it interacts with the CSL/RBP-Jκ transcription factor and its co-factor Mastermind (MAML1). The complex is tethered to promoters of Notch target genes. For details see the text.

The highly conserved Notch signaling pathway is critical for cell fate determination during development and tissue homeostasis [264,265]. It translates extracellular stimuli to transcriptional programs involved in cell cycle regulation and cellular differentiation [266,267]. The core architecture of this pathway is simple with only a few important canonical proteins (Figure 5) [268]. The human genome encodes five Notch ligands (Jagged 1/2 and Delta-like 1/3/4) and four Notch receptors (Notch 1–4). Notch ligands are transmembrane proteins expressed by various types of cells and tissues. Notch receptors are single-pass type I transmembrane proteins. They have a different amount of EGF-like repeats in the extracellular part. During maturation, these repeats are fucosylated by *O*-fucosyltransferase, and fucosyl moieties are further modified by the Fringe family of 1,3 *N*-acetylglucosaminyltransferases [269]. Such glycosylations represent “the code” which is responsible for specific recognition of ligands by different receptors. After glycosylation, the extracellular part of the Notch receptor is cleaved by the furin-like convertase (S1 cleavage) [270]. The non-covalently linked heterodimer is subsequently transported from Golgi to the cell surface. There are two additional domains between the membrane and EGF-like repeats—Lin20-Notch repeats (LNR) and heterodimerization domain (HD). Both are involved in ligand-dependent receptor activation.

Upon the receptor binding, the endocytic system is activated in the ligand-bearing cell. This forcefully drags the ligand-receptor complex towards the interior of this cell and, consequently, relaxes the structure of the LNR/HD domains. Once relaxed, the membrane-proximal region of the Notch extracellular domain (NECD) becomes a substrate for a disintegrin and metalloprotease 10/17 (ADAM10/17) metalloproteinases (S2 cleavage) [271,272]. They subsequently cleave NECD, which is then engulfed by a ligand-presenting cell via a transendocytosis [273]. The residual extracellular part is cleaved in the last proteolytic step (S3 cleavage). This is accomplished by γ-secretase, a membrane-bound protein complex involved in intramembrane proteolytic cleavage [274]. After this step, the Notch intracellular domain (NICD) is released to the cytosol and transported to the nucleus. The NICD has several domains. The N-terminally located proline–glutamate–serine–threonine-rich (PEST) domain is followed by a nuclear localization signal, seven ankyrin repeats, transactivation domain (TAD), and C-terminal RBP-Jκ–associated molecule (RAM). Inside the nucleus, NICD interacts via the RAM domain and ankyrin repeats with the CSL/RBP-Jκ transcription factor and its co-factor Mastermind (MAML1) [275,276]. In the absence of Notch receptor activation, CSL interacts with the co-repressor complex (CoR) [277,278]. This complex is tethered to promoters of Notch target genes, actively repressing them. The interaction with NICD and MAML1 leads to the displacement of the CoR complex and the recruitment of transcription co-activators (e.g., p300). This is followed by activation of target genes’ expression, including the Hairy enhancer of split 1 (HES1) family of transcriptional repressors, the CDK inhibitor p21, and others [266].

### 5.1. Notch Signaling Pathway and its Regulation by Ubiquitin Ligases

The Notch receptor is a short-lived protein which is targeted by multiple ubiquitin ligases. Several HECT-type ubiquitin ligases regulate its membrane localization and endocytic recycling, promoting “non-activated Notch receptor degradation”. Knockdown of the ITCH ubiquitin ligase leads to impaired Notch1 ubiquitination and lysosomal degradation [279]. Ubiquitin ligase ITCH interacts with the Notch receptor indirectly via the α-arrestin 1 (ARRDC1) and β-arrestins complex [280]. Interestingly, ITCH is also the main ubiquitin ligase involved in the NUMB-dependent Notch1 receptor inhibition [281]. Adapter protein NUMB, a major inhibitor of Notch signaling, is also a target of the RING-type ubiquitin ligase LNX2. This ubiquitin ligase, by mediating NUMB degradation, thus potentiates Notch signaling [282]. The WWP2 ubiquitin ligase was shown to mediate Notch1 polyubiquitination in a manner dependent on activated Dishevelled 2. Dishevelled 2 binds to the ubiquitin ligase WWP2 and unlocks its ligase activity from autoinhibition [283]. Of note, Dishevelled 2 involvement could point to possible signaling crosstalk between the Wnt and Notch1 pathways. Moreover, WWP2 also appeared in the screen for Notch3 interacting partners. It mediated ubiquitination of its active form and blocked Notch3 signaling in the context of ovarian cancer [284]. In general, ubiquitination of transmembrane receptors often regulates the receptor endocytosis and inappropriate activation. This was confirmed for Notch receptor. Its endocytosis is clathrin-dependent and requires epsin1 and the adaptor protein complex 2 (AP2) ubiquitin ligase Nedd4. Inactivation of Nedd4 leads to stabilization of membrane-bound Notch and signaling enhancement [285]. Another ubiquitin ligase involved in the regulation of the Notch cell surface stability is Deltex-1 (DTX1), which colocalizes with Notch1 on tubulovesicular recycling endosomes. Inactivation of DTX1 leads to Notch1 stabilization and cell surface relocation via the RAB4A-mediated transport route. Nevertheless, DTX1 does not mediate direct ubiquitination of the Notch receptor. The main DTX1 substrate in the endosomal compartment is PI5P4Kγ, a lipid kinase involved in PI(4,5)P2 production. It is PI5P4Kγ activity which is necessary for cell surface localization and stability of the Notch1 receptor [286]. The activated form of Notch (NICD) is also targeted by several other ubiquitin ligases, the most important being FBXW7. Phosphodegron-interacting protein FBXW7 belongs to the CRL family of RING-type ubiquitin ligases and recognizes the PEST region of NICD. This recognition is preceded by the sequence of priming and processing phosphorylations. The canonical priming kinase of the FBXW7 degron is CDK8, but integrin-linked kinase (ILK) is also able to phosphorylate it [287,288]. Like in other FBXW7 substrates, the processing kinase is GSK3β [289,290]. Moreover, the NUMB/ITCH complex and RNF8, a ubiquitin ligase involved in DNA damage response, are able to mediate NICD ubiquitination and degradation as well [281]. In support of RNF8 function as the Notch ubiquitin ligase, data from The Cancer Genome Atlas (TCGA) show an inverse correlation between RNF8 expression and Notch activity [291]. The ubiquitin ligase MDM2 targeting the p53 tumor suppressor plays an important role in potentiating the Notch signaling pathway via its interaction with Notch inhibitor NUMB. The mechanism remains to be elucidated [292,293].

Not only the receptors but also Notch ligands are regulated by ubiquitination. The ubiquitination is required for proper trafficking and presentation of the active ligands on the cell membrane and is provided by the E3 ubiquitin ligases Neuralized (NEUR) and Mindbomb (MIB) [273,294,295].

### 5.2. Mouse Models of Ubiquitin Ligases Involved in the Notch Signaling Pathway

Activation of the Notch receptor signaling pathway is important for embryonic development since it plays a critical role in cell fate determination. Expectedly, mice defective in this pathway often exhibit embryonic lethality and developmental abnormalities [296,297,298]. Several mouse models of ubiquitin ligases mentioned above confirm the role of these ligases in Notch signaling pathway in vivo. Mice deficient in Mib1 ubiquitin ligase clearly exhibit the Notch-defective phenotype, and mutant mice die early during embryogenesis with many developmental defects. On the other hand, mouse models of ubiquitin ligases which mediate degradation of many different proteins, for example FBW7, reflect this fact in their phenotypic complexity. Importantly, several mouse models do not support the importance of cognate ubiquitin ligases in the Notch signaling pathway, as discussed here [299,300,301].

#### 5.2.1. F-box and WD Repeat Domain Containing 7 (Fbxw7)

Human FBXW7 is a well-characterized F-box protein that binds to its substrates in a similar manner like β-TrCP. It is also a haploinsufficient tumor suppressor with mutations found in many human cancers [302,303]. It regulates the stability of many substrates involved in the cell cycle and survival, including p100, c-Myc, c-Jun, cyclin E, NF1, and Notch [304,305].

F-box and WD repeat domain containing 7-deficient mouse embryos die around E10.5–11.5. The phenotype clearly reflects that endothelial tissues represent a major site of embryonic Fbxw7 expression. The embryos have significant abnormalities in brain and yolk sac vascular development [306,307]. They also exhibit defects in major trunk veins formation and heart chamber maturation. The animals have the upregulated endothelial cell-specific isoform of the Notch receptors family, Notch4, as well as Notch target genes *HEY1* and *HES1*. Although the phenotype suggests a potential involvement of the Notch signaling pathway hyperactivation, no genetic rescue experiment has been done yet [307]. The T cell-specific deletion of *FBXW7* leads to thymic hyperplasia and subsequent development of the thymic lymphoma. T cells from knockout mice are immature, accumulate the Myc protein—another canonical FBXW7 substrate—and fail to exit from the cell cycle [308]. The targeted deletion of *FBXW7* in HSCs revealed the essential role of Fbxw7 in maintaining the HSCs pool. In Fbxw7-deficient animals, HSCs are prematurely depleted due to the fact of active cell cycling and p53-dependent apoptosis. The HSC reconstitution capacity and quiescence are impaired [309]. Mice with conditionally inactivated intestinal *FBXW7* develop a hyperproliferative phenotype. They show impairment in goblet cell differentiation and the accumulation of highly proliferating progenitor cells [310]. The brain-specific deletion results in perinatal death of embryos. Animals lack suckling behavior and have morphological abnormalities in the brain structure. On the cellular level, they have a clear impairment of neural stem cells differentiation, resulting in a decrease of mature neurons. They also have disequilibrium in neural cell differentiation towards astrogenesis [311]. The hepatic inactivation results in hepatomegaly and steatohepatitis. Mutant hepatocytes accumulate SREBP and NOTCH1 proteins. The long-term Fbxw7 deficiency leads to the proliferation of the biliary system and appearance of hamartomas as well as the imbalanced ratio between cholangiocyte and hepatocyte lineages [312].

#### 5.2.2. Mindbomb and Neuralized (Mib and Neur)

Mindbomb 1/2 proteins contain two substrate-recognizing domains—the ankyrin repeats domain and several RING domains. Neuralized 1/2 proteins consist of the neuralized homology repeats responsible for protein–protein interactions and the C-terminal RING domain. As described previously, Mib and Neur ubiquitin ligases target Notch ligands and influence Notch signaling.

Mindbomb 2-, NEUR1-, and NEUR2-deficient mice are viable with entirely normal appearance [299]. Mindbomb 1-deficient embryos are severely growth retarded at E9.5 and die at E10.5 from a lack of placental connection and defects in somitogenesis, vasculogenesis, and cardiogenesis [299,300,301]. The phenotype of these embryos clearly shows defective Notch signaling [296,313]. The yolk sacs have a blistered appearance with only small capillaries and complete lack of large vitelline-collecting vessels. Embryos lack heart looping and have an enlarged balloon-like pericardial sac and a smaller dorsal aorta. Other typical signs of Notch-related defects include irregular somitogenesis, absence of mesenchymal cells, and lack of second branchial arches. Knockout embryos show a strong neurogenic phenotype. The head of embryos appear normal but the neurons prematurely differentiate and undergo apoptosis. The reduction of progenitors leads to a loss of both astrocytes and oligodendrocytes. The embryos also lack intraembryonic hematopoietic progenitors [314]. Inducible inactivation of MIB1 (from E10 to E12), shows MIB1 continuous requirement in neuronal system development as it exhibits the suppression of glial differentiation [315].

The tissue-specific deletion of the *Mib1* gene shows its central role in Notch signaling and mouse development. Endoderm-specific inactivation causes a loss of endocrine progenitors and β-cells [316]. Its inactivation in the mouse myocardium mimics the phenotype of myocardial-specific deletion of *Jagged1*. Embryos have left ventricular non-compaction. They show reduced ventricular Notch1 activity, a dilated heart with a thin compact myocardium, and a large, non-compacted trabeculae protruding toward the ventricular lumen [317]. Mice with Mib1 inactivated in the bone marrow develop the myeloproliferative disease (MPD). They exhibit hepatosplenomegaly, accumulation of immature granulocytes, and anemia. Interestingly, the transplantation of wild-type bone marrow cells into the Mib1-null microenvironment results in a de novo MPD [318]. The absence of Mib1 during the development of the lymphatic system results in the developmental arrest of T cells and marginal zone B cells [319].

#### 5.2.3. Deltex-1 (Dtx1)

Deltex-1 is a RING-finger ubiquitin ligase containing the proline-rich motif and the N-terminal Notch-binding WWE domains. Deltex-1 regulates Notch signaling by controlling PI5P4Kγ stability [286]. It is downregulated in a subset of gastric adenocarcinomas [320].

Deltex-1-deficient mice have diminished Treg-dependent T cell anergy resulting in autoantibody production, augmented T cell activation, and increased inflammatory response [321]. The mice are otherwise healthy and fertile and T and B cell development seems intact [322]. Biochemically, Treg-initiated T cell anergy is dependent on the Foxp3 transcriptional factor. Deltex-1, which is transcriptionally activated by the nuclear factor of activated T cells (Nfat), controls Foxp3 activity via degradation of the FOXP3 inhibitor Hif1α. In Deltex-1-deficient Treg cells, the stabilized Hif1α suppresses FOXP3 and, subsequently, Treg’s ability to impose T cell anergy. Simultaneous knockout of Hif1α restores FOXP3 and rescues the defective suppressive activity in Deltex-1-deficient Treg cells in vivo [321,323]. It is not clear if Deltex-1 regulation of the Notch signaling pathway could be part of T cell anergy activation. As mentioned above, Deltex-1 is a positive regulator of Notch signaling [286]. Moreover, Notch was shown to act as the Foxp3 positive regulator [324,325]. Whether Deltex-1 activates Foxp3 also via potentiated Notch signaling or if Notch activation serves as positive feedback to sustain strong T cell anergy remains to be investigated.

#### 5.2.4. RING finger 8 (RNF8)

Ubiquitin ligase RNF8 has the C-terminal RING domain and the N-terminal forkhead-associated (FHA)-domain [326]. The FHA-domain is necessary for DNA-damage association. It binds to the ATM-phosphorylated N-terminus of the mediator of DNA damage checkpoint protein 1 (MDC1) [327]. Specifically, RNF8 targets histones by K63-linked ubiquitination, which is recognized by another ubiquitin-ligase RNF168 and leads to the recruitment of DNA repair proteins.

Transgenic embryos lacking Rnf8 are growth retarded with reduced hematopoietic populations. They have impaired class switch recombination and accumulation of unresolved immunoglobulin heavy chain-associated DNA double-stranded breaks. They are more susceptible to ionizing radiation, exhibit increased genomic instability, and have elevated risk for tumorigenesis [328]. Mouse males deficient in Rnf8 are sterile with defective ubiquitination of the XY chromatin. They are proficient in meiotic sex chromosome inactivation but deficient in global nucleosome removal [329]. Mutant mice also exhibit neuronal degeneration and reactive astrocytosis. Importantly, Rnf8-deficient neurons appear more susceptible to X-ray-induced DNA damage and Rnf8-deficient mice display memory impairment and reduced exploratory behavior in the open-field test. This defect could correlate with higher neuronal loss in these animals [330]. Cerebellar granule cell-specific RNF8 knockout displays a higher number of parallel fiber presynaptic boutons and functional parallel fiber/Purkinje cell synapses. It also revealed that RNF8 is involved in suppression of granule neuron/Purkinje cell transmission [331].

#### 5.2.5. Mouse double minute 2 (MDM2)

Mouse double minute 2 is a ubiquitin ligase with the C-terminal RING domain, the central acidic domain and the adjacent zinc finger region, and the N-terminal p53-binding domain that indicates its main function—to facilitate p53 ubiquitination and subsequent degradation. Despite some conflicting data, MDM2 was reported to be overexpressed in many different types of malignancies and it is usually related to a worse prognosis [332].

Mice with a hypomorphic allele of *MDM2* have defects in hematopoietic lineages. They develop mild anemia and the size of their lymphoid organs is significantly reduced due to the lower number of lymphocytes [333]. Mice with full inactivation of both *MDM2* alleles die early in development, and this phenotype is almost completely reversed with concurrent inactivation of murine *p53* [334]. Interestingly, mice with lowered levels of MDM2 were resistant to tumor formation, but otherwise were healthy and did not age prematurely [335].

## 6. Concluding Remarks

**Figure 6:**
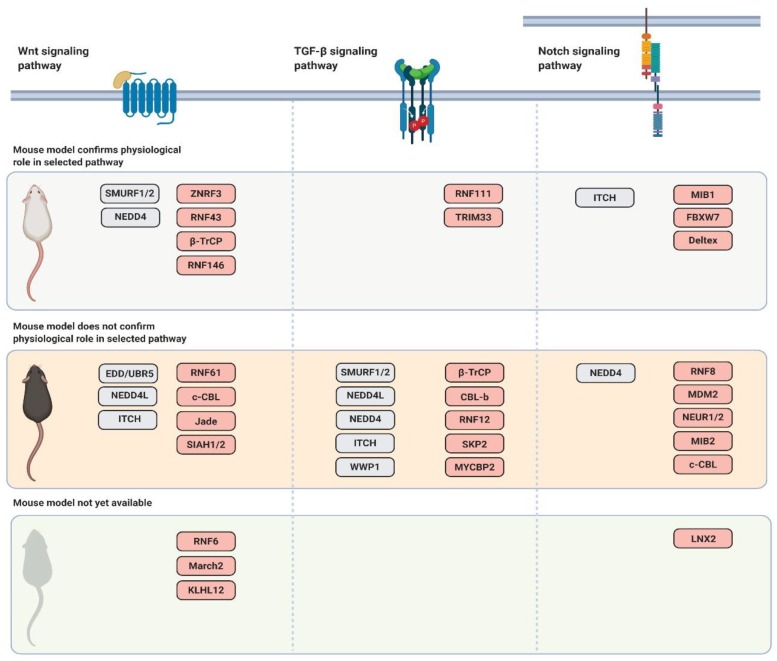
Schematic representation of mouse models of the selected ubiquitin ligases summarizing their physiological role in the Wnt, TGF-β, and Notch signaling pathways.

The importance of the ubiquitin–proteasome system has been emerging over the last three decades. Its discovery helped us to understand the biochemical nature of processes underlying major developmental and homeostatic events in the life domain. Each and every signaling pathway or cellular process depends on the UPS. The architecture of this system has both pleiotropic and specific facets. The pleiotropy is represented via proteasome while the specificity is ensured by a wide group of enzymes called ubiquitin ligases. Apparently, the UPS is essential for cellular and organismal homeostasis. This holds true especially for cancer cells which have to overcome the instability of the genetic information, and the control of the proteome is one way to do it. Therefore, they are fully dependent on proteasome function and this can be therapeutically exploited. The success of proteasome inhibitor bortezomib in multiple myeloma treatment fulfilled some of these expectations. Moreover, the re-discovery of thalidomide, a specific modulator of the ubiquitin ligase cereblon, for successful treatment of multiple myeloma initiated change of the focus towards the more specific approaches. It also proves that the right therapeutic options arise from fusion of the chemistry, the molecular biology, and the animal models. As presented in this review, there are numerous ubiquitin ligases which were found to be involved in the cancer-associated signaling pathways, but only few were confirmed to play the same role in vivo (Figure 6). Moreover, some of these ubiquitin ligases were shown to have a completely different function than expected. It is of the utmost importance to consider these observations and findings.

With the emerging technologies in genetic engineering, it should not be an option but a must to prove our results in mouse models. Because these models will not only confirm what we think we achieved in Petri dishes, they will be an important part of the next step—how to translate these results into better cancer therapy.
